# Influenza A Virus Entry: Implications in Virulence and Future Therapeutics

**DOI:** 10.1155/2013/121924

**Published:** 2013-01-09

**Authors:** Emily Rumschlag-Booms, Lijun Rong

**Affiliations:** ^1^Department of Biology, Northeastern Illinois University, Chicago, Chicago, IL 60625, USA; ^2^Department of Microbiology and Immunology, College of Medicine, University of Illinois at Chicago, IL 60612, USA

## Abstract

Influenza A viruses have broad host tropism, being able to infect a range of hosts from wild fowl to swine to humans. This broad tropism makes highly pathogenic influenza A strains, such as H5N1, potentially dangerous to humans if they gain the ability to jump from an animal reservoir to humans. How influenza A viruses are able to jump the species barrier is incompletely understood due to the complex genetic nature of the viral surface glycoprotein, hemagglutinin, which mediates entry, combined with the virus's ability to use various receptor linkages. Current therapeutics against influenza A include those that target the uncoating process after entry as well as those that prevent viral budding. While there are therapeutics in development that target entry, currently there are none clinically available. We review here the genetics of influenza A viruses that contribute to entry tropism, how these genetic alterations may contribute to receptor usage and species tropism, as well as how novel therapeutics can be developed that target the major surface glycoprotein, hemagglutinin.

## 1. Introduction

Influenza viruses belong to the Orthomyxoviridae family, which consists of several genera. The first includes both influenza A and B viruses, while another is comprised of influenza C virus [1]. These classifications are based on the distinct antigenic nature of the internal nucleoprotein and matrix proteins of each virus. Infection with influenza subtypes B and C is mostly restricted to humans [2, 3], while subtype A is able to infect a wide range of hosts including but not limited to humans, swine, horses, domestic and wild birds, fowl, and dogs [4–8]. This broad spectrum of hosts plays a pivotal role in the ability of the virus to reassort, mutate, and spread, all of which contribute to the ever-present global threat of influenza. 

 Influenza A virus poses the most serious hazard of the three subtypes, causing global economic losses as well as severe health concerns. Influenza A virus is the causative agent of severe respiratory illness infecting nearly 15% of the world's population with upwards of 250,00–500,000 deaths estimated by the World Health Organization. Infections are characterized by upper respiratory distress along with high fever, myalgia, headache and severe malaise, nonproductive cough, sore throat, and rhinitis. Severe illness and death are mainly associated with the young, elderly, and those with compromised immune systems [2]. 

Influenza viruses have ravaged human and poultry populations around the world for centuries, causing serious illness and death, major economic loss, in addition to instilling fear as the next potential deadly pandemic. During the twentieth century, this virus caused three major pandemics, which resulted in an estimated 20–50 million deaths combined worldwide [9–11]. In the twenty-first century, 2009 Pandemic H1N1 was caused by a reassorted swine strain. The reassortment included influenza viruses of human, avian, and two swine strains [12]. The resultant reassorted swine strain then jumped to humans, spreading around the world within a few weeks [12, 13]. The initial result of this event was more than 22 million reported cases, 13,000 deaths, the blocking of countries' borders, and the closing of numerous schools [14]. A recent study suggests the actual impact may be more than 10 times the initial estimates [15]. While we are currently in the postpandemic phase, this H1N1 strain is the currently circulating endemic influenza strain among human populations. Upwards of 20–40% of the world's population is thought to have immunological protection for the time being, as they have already been exposed to the virus. 

Influenza viruses possess several unique characteristics, many of which potentiate the menace posed by this virus. One such feature is the segmented nature of the viral genome [16]. The virus carries eight negative-sense RNA segments. Due to the segmented nature of the viral RNA, if a host cell is infected with two viruses of different influenza strains, the gene segments of one virus can recombine with those of another virus during replication. This reassortment event is referred to as antigenic shift. The newly formed virus can be especially dangerous if a human adapted strain acquires gene(s) which transform it into a highly pathogenic strain, or if a highly pathogenic strain acquires the necessary gene(s) to infect and spread amongst humans. Either scenario is predicted to raise serious threats worldwide, as was the case in 1957 and 1968 [17, 18]. 

 The major influenza pandemics in the twentieth century, along with the 2009 Pandemic H1N1, are thought to have arisen via antigenic shift. The pandemic of 1957, better known as “Asian Influenza” H2N2 virus, was originated in Southern China and spread rapidly to the United States and Europe causing more than 1 million deaths worldwide [19]. Sequence analysis along with biochemical studies suggest that this particular virus was originated from the reassortment or genetic mixing of an avian virus with that of a human virus [19–22]. While the recombinant virus was not particularly virulent, the high level of mortality associated with it is attributed to the immunological naivety of the infected populations. A similar scenario was seen with the pandemic of 1968, the “Hong Kong Influenza.” The HA gene of this virus was of the H3 subtype and originated from an avian source along with the PB1 viral polymerase protein [21, 23–25]. These two avian gene segments reassorted with a human virus, creating a new virus with greater virulence and the ability to infect humans. Furthermore, human populations were immunologically naïve to this recombinant virus, making the health impact that much greater. Much devastation and loss are attributed to pandemics arising from antigenic shift and it is antigenic shift that is predicted to be the likely cause of the next pandemic [26]. Furthermore, evidence points to antigenic shift as the perpetrator of the most severe of the influenza pandemics. It is believed that antigenic shift was responsible for the first and most severe pandemic of the 20th century in 1918, killing an estimated 50 million people worldwide [27–32]. Recent sequence analysis of this H1N1 virus, referred to as the “Spanish Flu”, strongly suggests that the virus was directly transmitted to humans from an avian source [32]. 

While antigenic shift is a powerful means of acquiring genetic change, antigenic drift results in more subtle changes in the genome. Antigenic drift in influenza viruses refers to residue substitutions in the virus' coding sequence via point mutations [33]. Due to the lack of a proofreading function in the RNA polymerase, influenza constantly accumulates mutations within its genome during replication. These mutations may be silent or they may alter the virulence and pathogenicity of the virus. For instance, if a highly pathogenic avian virus acquires the necessary mutations that facilitate its ability to efficiently enter and replicate in humans, then the virus can become a serious threat to humans. 

The viral surface is studded with two major surface spike glycoproteins, hemagglutinin (HA) and neuraminidase (NA), which differ greatly in genetic variation [8]. In addition, an essential ion channel protein, M2, exists on the virion surface. HA and NA exist on the virion surface in a ratio of approximately 4/5 : 1, with an estimated 400–600 total spikes. HA is responsible for mediating entry into target cells via the host cell receptor, sialic acid (SA). NA plays a major role during the budding process by releasing progeny virions from the host cell. To date, 16 subtypes of HA and 9 subtypes of NA have been identified [34, 35]. These subtypes have been mainly identified amongst different avian species, as birds are the natural reservoirs of influenza virus [7, 8]. Since entry is the first requirement for infection, it is crucial that we understand its role in host tropism, pathogenesis, as well as the role of differences between HA subtypes and species-specific viruses. Furthermore, HA has garnered recent attention as a target for broad-spectrum neutralizing antibodies [36, 37].

 HA has been shown to be an important determinant for influenza virus virulence and pathogenesis. Genomic studies of the 1957 (H2N2) and 1968 (H3N2) pandemics revealed that a major contribution to virulence was due to the exchange of the HA segments between human and avian strains [24]. Sequence comparison of the 1918 (H1N1) virus to other influenza A viruses from various species reveals that the entire 1918 virus is more closely related to avian influenza A viruses than with any other species, namely humans, suggesting that accumulated mutations in the avian HA gene allowed it to better adapt to the human host. The critical role of mutations within the avian virus genome underlies the importance of studying mutations within the H5N1 virus genome that may be critical to sustain infection in and among humans although no sustained human-to-human transmission has been reported yet [38, 39]. 

HA exists on the virion surface as a trimer of HA_1_ and HA_2_ subunits linked by disulfide bonding. This surface glycoprotein is first synthesized as a single polypeptide (HA_0_) of approximately 550 amino acids, which is highly N-glycosylated. HA assembles into a trimer in the rough endoplasmic reticulum (ER) before passing through the Golgi complex on its way to the cell membrane. For the virus to be infectious, the HA_0_ precursor protein must be cleaved into its subunits, HA_1_ and HA_2_ [40, 41]. If HA is not cleaved, fusion of the viral envelope with the endosomal membrane cannot occur, thus the genomic contents cannot be released within the target cell. At the structural level, cleavage of HA_0_ is important because it reveals the hydrophobic N-terminus of HA_2_, the fusion peptide, which is inserted into the host membrane during HA-mediated viral/host membrane fusion and viral entry. Upon endocytosis, the acidic pH (5–5.5) environment triggers HA_2_ to undergo the irreversible [42–45] conformational changes necessary for fusion to occur, allowing the viral and host membranes to fuse, releasing the viral genomic contents into the cytoplasm to begin replication [46]. 

 Two known classes of proteases are involved in HA_0_ cleavage [47–49]. The first known protease class recognizes a single arginine at the cleavage site. HA with such a cleavage site is processed at the cellular surface during the budding process or on released viral particles by secretory proteases, such as tryptase Clara, a trypsin-like protease found in the alveolar fluid of rat lungs, plasmin, and bacterial proteases [50]. This particular set of trypsin-like enzymes is found either in specialized cells or within specific organs, thus viruses carrying such an HA have more restricted activation, infection capability, and therefore limited replication and spread [40, 41, 50]. Due to this restriction, trypsin-like activated viruses are generally thought to be less pathogenic. It is interesting to note that while low pathogenicity is generally associated with a virus whose HA has the trypsin-like cleavage site, the most highly pathogenic human influenza virus was restricted to trypsin-like enzyme cleavage [51]. The enzyme-limited, restricted sites of replication correspond with sites of natural infection for humans and birds, that being limited to the upper-respiratory tract (humans) or gastrointestinal tracts (birds) [20]. 

 The other variant of HA contains a polybasic consensus sequence cleavage site, R-X-K/R-R, which is recognized by the subtilisin-like endoproteases, furin, and PC5/6 [52, 53]. This protease is expressed in the trans-Golgi network, therefore the HA is activated during the exocytic route during virus maturation [53, 54]. As this protease is nearly ubiquitously expressed, a virus with this cleavage site can replicate throughout a host and cause extensive systemic spread. Due to this characteristic, viruses with the polybasic site are generally considered highly pathogenic strains, such as the H5 and H7 strains [53, 55]. A comparative analysis at the entry level between a low pathogenic H5N2 strain and a highly pathogenic H5N1 strain revealed that the major restriction to entry was the cleave site sequence. Replacement of the monobasic cleavage site into the polybasic cleavage site on HA enhanced the HA-mediated entry [56]. However, the worst human influenza epidemic recorded, the 1918 Spanish Flu, did not have the polybasic cleavage site as previously discussed. Instead, it had a single arginine, highlighting the complicated nature of influenza viruses and their virulence and pathogenicity [51]. 

In addition to the aforementioned two classes of proteases, the following proteolytic enzymes are implicated in HA activation: type II transmembrane serine proteases (TTSPs) TMPRSS2, TMPRSS4, and human airway trypsin-like protease (HAT) [47]. While less is known about these proteases, their activity may be additionally associated with viral pathogenicity and tropism. 

 Influenza entry tropism is mainly determined by the binding preference of HA_1_ to its receptor, SA. SA has long been believed to be the sole receptor for the influenza virus. It was discovered nearly 70 years ago that upon addition of influenza virus to chicken erythrocytes, the cells would agglutinate at 4°C [57]. A shift in temperature to 37°C would cause the virus to elute, while addition of new influenza virus no longer caused agglutination. This phenomenon suggested that, in addition to binding a surface molecule on the erythrocytes, the virus carries a receptor-destroying enzyme. It was later discovered that the cellular component removed by the virus was SA and that treatment of erythrocytes with purified sialidase from *Vibrio cholerae* prevented agglutination [58–60]. This finding was the first demonstration that SA acts as a receptor for influenza A viruses. 

 SA encompasses a large family of sugar molecules. The most prevalent member of this family is N-acetylneuraminic acid (NeuAc). It primarily exists as a six-carbon ring with several unique components extending from the ring. The most important feature of SA, with regards to influenza, is the manner in which the free sugar is attached to the host cell surface. Host cells carry various surface glycoproteins and glycolipids, many of which are highly modified. These surface proteins that are modified with a terminal SA play a crucial role in influenza entry, serving as the viral attachment and entry receptor. SA can be attached to the underlying glycocalyx in one of three main linkage patterns, either *α*2,3, *α*2,6, or *α*2,8 [61]. While other linkages exist, these three are the most prevalent in mammalian cells [62]. 

 In addition to viral entry, SA plays an equally important role in determining host tropism. Influenza tropism is highly influenced by the linkage of SA, with avian and human viruses preferentially utilizing different linkages. Avian viruses have been classified as predominantly *α*2,3 specific, while human viruses tend to favor the *α*2,6 linkage [63–66]. These preferences have been established from studies examining SA distribution within a specific host, binding of the virus to SA at the protein level, as well as studies analyzing replication efficiency in the presence of these specific linkages. Increased prevalence of the *α*2,3-linked SA in the avian gastrointestinal tract correlates with the ability of the virus to enter and replicate here, as it is the site of natural infection for birds [23, 61]. As the natural carrier of the virus, avian populations usually display few disease symptoms [7]. Due to the high prevalence of receptors within the gastrointestinal tract, the virus replicates most efficiently there and is excreted through waste products. On the other hand, human influenza viruses predominantly utilize SA in *α*2,6 conformation which is highly prevalent in the upper respiratory tract [23, 65, 67, 68]. SA linkages within the human respiratory tract are present on nasal mucosal epithelial cells, paranasal sinuses, pharynx, trachea, and bronchi, all carrying *α*2,6 SA while *α*2,3 SA is found on nonciliated cuboidal bronchi and alveolus, as well as type II cells within the alveolar wall [69]. Based on these data, it is the low levels of *α*2,3 linkages in the upper tract that have been postulated as being a block for efficient infection and replication of *α*2,3 preferring viruses in humans. On the other hand, flow cytometry studies using lectins specific for *α*2,3 and *α*2,6 SA showed that both linkages were present on human bronchial epithelial cells with *α*2,6 SA being the vast majority [70, 71], suggesting another layer in the complexity of influenza tropism beyond SA preference. In addition, it still remains unclear if all subtypes of influenza virus target the same subset of respiratory cells and/or have the same affinity and avidity for *α*2,3 and *α*2,6 SA linkages. Besides terminal sialic-acid linkages, specificity is also influenced by internal linkages along with modification of inner oligosaccharides including sulfation, fucosylation, and sialylation [72, 73]. Overcoming this binding restriction may be one step needed for avian influenza to more efficiently spread from human to human. 

 The linkage of SA and its influence on influenza entry has been extensively characterized to determine its role in tropism; however in early 2008 an additional level of complexity was revealed. Chandrasekaran et al. reported that the crucial determinant for influenza tropism is the structure of the underlying glycocalyx, not the terminal SA linkage [74]. Using a series of analyses, it was reported that avian viruses prefer SA in a cone-like topology. This shape is adopted by SAs, both *α*2,3 and *α*2,6, with short underlying glycan(s) and allows HA to contact Neu5Ac and galactose sugars in a trisaccharide motif. Human viruses are reported to prefer an umbrella-like glycan topology, which is unique to *α*2,6 SAs with long underlying glycans. This report also concluded that *α*2,6 alone is insufficient for human transmission, as avian viruses can utilize *α*2,6 SA on a short sugar chain, suggesting that the virus must adopt the ability to utilize *α*2,6 SA on a long sugar chain. It was concluded that it is the glycan composition, and thus the SA topology that may influence influenza tropism, not just the SA linkage present. 

The region of HA that is responsible for binding SA is referred to as the receptor-binding domain (RBD). This pocket-shaped depression is located at the membrane-distal tip of each monomer within the trimeric HA structure and is comprised of the 190 helix (residues 190–198), the 130 loop (residues 135–138), and the 220 loop (residues 221–228) based on H3 numbering [25]. Several key conserved residues including tyrosine 98, tryptophan 153, and histidine 183 form the base of the binding pocket and are crucial for maintaining the structural basis of the binding pocket as well as in forming interactions with SA [75]. Sequence analysis from several strains of influenza along with structural modeling has given great insight into the residues that play a pivotal role in SA binding preference [25, 76]. 

 Structural studies of HA suggest that avian and human influenza viruses appear to be distinct in their RBDs at positions 226 and 228 [77, 78]. Avian HAs tend to have glutamine and glycine at the respective positions while human HAs carry leucine and serine [78–81]. The avian HA with these residues forms a narrow binding pocket for the *α*2,3 receptor while the change in residues for human HAs results in a broader pocket for the *α*2,6 receptor. Neither position 226 nor 228 plays a direct role in binding, rather they seem to influence the contour of the pocket [70, 82]. These differences in pocket shape correlate well with the glycan topology studies. Additionally, it was shown that a lab-adapted strain which prefers *α*2,6 binding was able to switch preferences to *α*2,3 when grown in the presence of *α*-2 macroglobulin, a *α*2,6 glycoprotein found in high concentrations in horse sera [65]. Based on these and other studies, it seems that residue 226 and 228 have an important indirect role in SA binding compatibility and therefore preference. 

 Interestingly, the 1918 H1N1 virus HA carried glutamine 226 and glycine 228 corresponding to the avian *α*2,3 receptor, however the virus is able to bind the *α*2,6 receptor, demonstrating that changes at these positions are not necessary for altered receptor binding [30]. Further analysis of the 1918 HA revealed that a single change from asparagine to glutamate at position 190 was responsible for the altered binding phenotype [30]. In addition, a change from asparagine to glycine at 225 in combination with the change at 190 increased respiratory transmission of the virus in the ferret model, further highlighting the importance of the RBD residues. 

 More extensive studies focusing on the H3 subtype revealed that human viruses with *α*2,6 preference have leucine at 226 and serine at 228, while avian viruses with *α*2,3 preference have glutamine at 226 and glycine at 228 [63, 82]. Residues 193 and 218 have also been implicated as important determinants for receptor preference in the H3 subtype, however specific residue changes have not been fully established [83]. 

 Similarly, studies focusing on seasonally circulating H1 viruses found avian and human viruses differ at two positions in their binding preference. A proline at 186 and a glycine at 225 correspond with *α*2,3 type binding, while serine 186 and asparagine 225 are favored by *α*2,6 type binding [25]. Interestingly, HA proteins of both avian and human viruses have glutamine at 226 and glycine at 228. The discrepancies highlighted by the H3 and H1 studies in combination with the studies on the H1N1 Spanish Flu illustrate the complex nature of receptor binding, that is, not all influenza A viruses behave in a similar way, not even among avian strains nor human strains. 

 Since the initial H5N1 influenza outbreak that began in China in 1997, several studies have focused on the RBD of this particular viral strain. These studies include the use of sialoglycoconjugates, crystal HA structures, and simulated computer-based binding assays [55, 69, 72, 74, 84–87]. Residues that have been implicated in human receptor-type binding include asparagine 227, asparagine 159, lysine 182, and arginine 192. It is proposed that residues 159, 182, and 192 influence binding by stabilizing the HA binding pocket and maintaining structural integrity, as these residues are not in direct contact with SA. A switch of glutamine to leucine at position 226 and a switch of glycine to serine at position 228 equates a shift from avian receptor to human receptor specificity, as seen in the H3 virus as well [86, 88]. Further studies specifically targeting the HA of the A/Vietnam/1203/2004 H5N1 virus demonstrate the importance of mutation E190D which reduced the binding to 2,3 linkages, as well as the double mutant Q226L/G228S [86]. In addition the H5N1 HA surface residue tyrosine 161 has recently been implicated in altering glycoconjugate recognition and cell-type dependent entry. Substitution of tyrosine 161 to alanine switched the binding preference from N-acetylneuraminic acid to N-glycolylneuraminic acid [87]. It is important to note that different strains of the H5N1 virus from different time points during the virus outbreak were used in these studies.

To better understand the role of naturally acquired mutations and their ability to potentiate sustained transmission, two recent studies showed that as few as four to five amino acid substitutions along with gene reassortment may be sufficient for respiratory droplet transmission between ferrets [89, 90]. Herfst et al. identified (based on H5 numbering) glutamine to leucine at 222 and glycine to serine at 224 as critical residues to alter sialic acid specificity from the avian *α*2,3 SA to the human *α*2,6 SA. Two additional HA substitutions, threonine to alanine at 156 and histidine to tyrosine at 103 play a role in disrupting N-linked glycosylation and monomer interaction, respectively. Lastly, a switch from glutamate to lysine at 627 in the polymerase PB2 subunit was identified, which is a common change seen in mammalian adapted influenza strains. Imai et al. identified four substitutions, all within HA. Similar to the results of Herfst et al., it was found that substitutions of asparagine to lysine at 220 and glutamine to leucine at 222 can alter SA specificity from the avian *α*2,3 SA to the human *α*2,6 SA. Again, a disruption in N-linked glycosylation via asparagine to aspartate at position 154 was identified along with a change in the stalk region corresponding to threonine to isoleucine at 315.

 While the linkage of SA and the residues within the RBD plays a significant role in viral entry, the glycoconjugate to which SA is attached appears to be an additional important factor. Chu and Whittaker determined that cells deficient in the GnT1 gene lack terminal N-linked glycosylation, rendering them deficient in influenza A viral entry [91]. The GnT1 gene encodes the enzyme N-acetylglucosaminyltransferase, which is involved in modification of N-linked glycans in the Golgi apparatus. These cells lack N-glycoproteins, but still possess surface *α*2,3 SA and *α*2,6 SA. While considering this phenotype, this mutant Chinese Hamster Ovary (CHO) cell line has the capacity to bind HA and expresses sufficient levels of both SA linkages on the cell surface for attachment. In this mutant CHO cell line, complete entry was blocked, as virions were not endocytosed. These results suggest a specific role for N-linked glycoproteins in influenza A entry, perhaps acting as a cofactor in mediating entry. 

 There is evidence suggesting that SA is not necessary for infection by influenza. The HA of a human H1N1 strain was shown to bind glycoconjugates other than SA [92]. In addition, Stray et al. demonstrated the ability of desialylated Madin Darby canine kidney (MDCK) cells to be infected by influenza A virus [93]. A study by Nicholls et al. demonstrated the ability of the H5N1 virus to infect upper and lower respiratory tract cells in the presence or absence of *α*2,3 SA which is believed to be the entry receptor for this virus [94]. In addition, the levels of SA on susceptible and nonsusceptible cells do not correlate with either *α*2,3 SA or *α*2,6 SA for an H5N1 strain [95]. Taken together, it is possible that the barrier to efficient human infection and human-to-human transmission of the H5N1 virus is not due to SA linkages, but rather it is due to inefficient use or expression of a yet unidentified entry mediator. 

 To fight the spread of influenza, prophylactic therapeutics and vaccines continue to be vital methods of control. Vaccines are the primary means of controlling the spread of the virus and are based on inactivated viruses, live attenuated viruses, or purified viral protein(s) that illicit a strong neutralizing antibody response [96]. Of these vaccines, most target the globular head region of HA, containing the receptor-binding domain, thus preventing attachment of the virus to susceptible cells. These neutralizing antibodies are rarely immunoresponsive to an alternate influenza strain, often losing their potency as their corresponding strain acquires mutations during circulation. Furthermore, due to the virus ability to constantly acquire genetic changes, it is difficult to predict what the circulating strain(s) for the upcoming year will be. A mismatch of vaccine strain with circulating strain(s) will offer little to no protection. 

In addition to vaccines, anti-flu therapeutics have been developed which can be divided into two classes, anti-NA and anti-M2 [97]. The first class of inhibitors specifically targets the NA protein of the virus, halting the spread of progeny virions [98]. During the budding process of influenza, newly produced progeny virions are tethered to the host cell surface via HA proteins interacting with SA molecules. NA functions in recognizing this HA-SA interaction and cleaves the SA moiety, releasing the viral particle [99]. The currently approved therapeutics for influenza infection include NA inhibitors which block this step, thus preventing release and further spread of the virus, both within the infected host and consequently to others. Included in this category of antivirals are two of the most commonly used therapies, Zanamivir (trade name Relenza) and oseltamivir phosphate (trade name Tamiflu) for the treatment and prevention of influenza A and B viruses. Zanamivir and oseltamivir phosphate are competitive inhibitors for the active site of the NA enzyme [100]. While Zanamivir and oseltamivir phosphate can be highly effective in both treating influenza and in outbreak control, in the 2008-2009 flu season, nearly 100% of H1N1 samples tested by the Center for Disease Control (CDC) were shown to be resistant to oseltamivir phosphate [101, 102]. This high level of resistance highlights the need to develop new antivirals against influenza. 

 Another class of influenza inhibitors, the adamantanes, is those which block the M2 ion channel on the virion surface. The M2 ion channel is embedded within the virion lipid bilayer and facilitates hydrogen ion transport, ultimately leading to virion uncoating and replication during the entry process [103]. The adamantane derivatives, amantadine and rimantadine, were approved for the treatment and prevention of influenza A only, as only influenza A class viruses have the M2 protein [103]. The CDC and WHO report that greater than 99% of circulating strains are resistant to M2 inhibitors and have recommended their use to be discontinued. Therefore these inhibitors are only used when a specific nonresistant strain is thought to be the causative infectious agent [101]. These antivirals, along with the NA inhibitors, provide a treatment option after infection, however since not all influenza A viruses respond to these treatments, these drugs may be ineffective. In addition, resistance to these treatments over the course of an outbreak or influenza season underlies the urgency to develop new antiviral therapies. 

 Since there is a lack of clinically available inhibitor(s) which targets the major viral surface protein, HA, recent clinical trials are pushing forward to expand the repertoire of influenza therapeutics, including drugs that target HA, which is the target of most neutralizing antibodies [104]. These new drugs include cyanovirin-N and thiazolides. Cyanovirin-N targets the high-mannose oligosaccharides, neutralizing the viral particle [104, 105]. The thiazolides work to block the maturation of HA posttranslationally [104, 106]. Recently, several groups have identified broad-spectrum neutralizing antibodies that are protective against group 1 (which includes HA subtypes 1, 2, 5, 6, 8, 9, 11, 12, 13, and 16) and group 2 (which includes HA subtypes 3, 4, 7, 10, 14, and 15) influenza A viruses [36, 107–109]. These broad-spectrum neutralizing antibodies (referred to as CR6261, F10, and F16) are further distinct in that they target the stem region of the HA molecule. The proposed model is that by targeting the stem region, more specifically the fusion peptide, these antibodies are able to prevent exposure of the fusion peptide under acid conditions. This trapping mechanism prevents the viral membrane from fusing with the host endosomal membrane, locking the genetic contents within the viral particle. An additional stem-directed neutralizing antibody, CR8020, is active against group 2 influenza A viruses. While not as broad in activity as the aforementioned antibodies, CR8020 could be utilized in conjunction with a group 1 neutralizing antibody, providing a one-two punch against both HA subunits.

 Influenza viruses will continue to circulate among animal and human populations, acquiring and exchanging genetic components as they do. Due to the constant change in their genetic profiles, influenza viruses will continue to pose a serious threat, from both an economic and public health point of view. Continued study and surveillance of influenza viruses is further highlighted by our inability to maintain effective influenza vaccines and prophylactic therapeutics. The prospective of new antivirals, especially those that provide broad-spectrum protection, provide renewed efforts in our ability to control influenza infection and spread.
